# Water—Its Relation to Health and Disease

**Published:** 1880-01

**Authors:** Wooten M. Wilkerson

**Affiliations:** Marion, Ala.


ARTICLE II.

### WATER—ITS RELATIONS TO HEALTH AND DISEASE.

BY WOOTEN M. WILKERSON, A. B., M. D., MARION, ALA.

It has often been a subject of remark, how little attention is paid to the hygienic surroundings of our people, especially as regards the hygiene of water. So great is this ignorance and neglect, that whole families and communities often suffer the direst epidemics, and numbers are carried off by the use of water containing poisons of the worst type. It is not strange that these disastrous results do follow, when we consider the intimate relations which this fluid bears to the body and its vital organs. This inorganic material is present in the body in larger quantities than any other substance, constituting at least three-fourths of its entire weight. It is present in every organ, and pervades every tissue, except, perhaps, the enamel. It is the great solvent, and thus the vehicle for the nourishment of the body as well as for the removal of its excrementitious substances. By means of it the nutrient material taken into the alimentary canal is dissolved, absorbed, and carried to the remotest parts of our organism for its supply, and by it the excrements are brought from these remote parts to find their egress from the body by means of certain organs.

Water is a necessary constituent of the body, and when taken in proper quantities and of sufficient purity can only be conducive to health. From sixty to eighty ounces are consumed daily by a healthy man of average size, though this may vary greatly according to individual peculiarities, some taking much less. From experiments on the lower animals it is found absolutely necessary that this amount should be maintained, otherwise a peculiar sensation, known as thirst, comes on, from which the animal dies in a few days, generally four or five, regardless of the amount of food given. If, on the contrary, all food be stopped, and water alone be given, the animal will continue to live for twelve or thirteen days, and finally die of emaciation, surviving thrice the length of time on water alone that it did on food. Thus, it is shown that this fluid plays an important part in the economy of our bodies, for it may administer to our life, or may snatch from us that precious boon, owing to its intimacy with the vital parts and the poison it may contain.

That it should carry out fully its mission as a health agent, it must be freely applied externally. That bathing the whole body daily is conducive to health cannot be denied. But this is not always practicable; yet it should be indulged in as frequently as possible—certainly not less than two or three times a week, and at other times as much of the surface as is convenient should be thoroughly cleansed. Neglect of these precautions is the source of many annoying diseases, especially of the skin. Tumors, too, are liable to be formed by the accumulation of sebaceous matter in the glands, the outlets of which have been closed through neglect of proper ablutions. If water be used freely, the skin will assume a healthy glow, capillary circulation will be increased, and the sudoriferous glands will be kept well open, allowing the rapid removal of the perspiration and its excrements. This relieves the system of a large amount of effete and poisonous material; hence the healthy feeling subsequent to a bath.

As regards health, we are mainly concerned with water for drinking purposes. For this, as pure a water as can be obtained should be used. The water taken into the stomach is absorbed, and permeates the whole frame to its minutest parts. Should it contain any impurities, these are borne along with the general current, and bring their poisonous properties to bear directly on the vital organs. The water from all sources varies greatly in composition, yet all of it, when within certain limits, may be freely used without detriment to the health. As a matter of fact, no water is found that is absolutely free from unwholesome properties.

Rain water is the purest furnished by nature, though it contains impurities of several kinds. In it are found such impurities as may be in the atmosphere at the time of its falling; such are carbonic acid, ammonia, nitrogen, and any organic matter which may be present. This water, however, is not generally used, except in certain localities where water is scarce, and is then kept in cisterns, which render it liable to other sources of infection.

Surface and subsoil waters are likely to be exceedingly poisonous. For, running over soil containing decaying vegetable or animal matter, as manures, leaves, etc., it dissolves large quantities of nitrites, nitrates, and any soluble salts that the soil may contain. This renders it particularly unfit for use, and should never be used when possible to avoid it. These waters, from standing in ditches and shallow wells, are apt to contain animal or vegetable germs of an exceedingly poisonous type, adding one other serious objection to their use.

Marsh water is excessively poisonous, and should never be used. It contains large quantities of decaying vegetable and some animal matter. Its odor is sufficient to testify to its unwholesomeness, and assists us in putting a veto on its use.

Well and spring waters are the two most commonly used for drinking purposes. The compositions of these are as various as are the localities in which they are found. All of them contain salts of some kind in solution, and are more or less salubrious, according to the kind and quantity of these salts. They may contain salts that are not soluble in water alone, but are dissolved by means of the large amount of carbonic acid contained in the water. For, as it permeates the earth, it dissolves a large amount of this gas, the interstices of the soil containing 250 times as much as the air. These waters may also contain organic matter of various kinds, depending on the presence of this matter in the neighborhood. It must be remembered that a well drains an area, of which it is the centre, with a radius of 100 yards at the surface of the earth. This forms a truncated cone with its base upward, the bottom of the well being its apex. A portion of all organic matter contained within this space, will be dissolved by the water as it permeates the earth, and will be found in the water of the well. Hence no abundant source of organic matter should be allowed to remain within this area—such as water-closets, manure heaps, slop-deposits, etc. If absolutely necessary that they should be in close proximity to the water supply, they should be provided with closely-fitting sewers, which will convey the refuse beyond the space above specified. If this is impossible, the closest attention should be given to cleanliness by the frequent use of suitable disinfectants. Otherwise much mischief may result from the accumulation of poisonous matter in the system, deposited there by the drinking water.

River water is much used for drinking purposes. Its composition varies greatly according to the river from which it is taken, and these of course with the soil over which it flows and the impurities along its banks. Nearly all contain more or less unhealthy matter, though some are comparatively free, as the river Loka, in Sweden, which is said to contain only $\frac{1}{25}$ of a grain of mineral matter to the gallon, flowing, as it does, over insoluble granite. River water is often contaminated with the sewage of towns, cities and manufactories along its banks. From these sources it may be excessively poisoned by organic matter, though a large part of this will disappear by oxidation, due to constant exposure to the air. Hence, if sufficiently removed from the source of contagion, this water may be used with impunity.

Thus we find that no water supplied by nature is free from impurities; and the question will naturally be asked, "What standard of purity shall be used?" In answer to this question, no positive rule can be given. But Parke's classification is the best guide, the substance of which is given below:

1. *Pure* and *wholesome* water should be clear, inodorous, aerated and sparkling, and should not contain more than 8 grains of solid matter to the gallon, of which only one should be dissipated by heat, and, presumably, organic. If it be a chalk water, there may be 14

grains of calcium carbonate. Must be no nitrites, and mere traces of nitrates and ammonia. The albumenoid ammonia should not exceed the .0056 of a grain per gallon.

2. *Usable water* should be clear, inodorous, aerated and sparkling. Solids should not exceed 30 grains to the gallon, and these either sodium or calcium carbonates. Sulphate and chloride of sodium are permissible, with a little calcium and magnesium sulphate. Reactions of nitrites, nitrates and ammonia must be inconsiderable. Albumenoid ammonia should not exceed .007 of a grain per gallon. If the saline ingredient be a mixture of carbonate and chloride of sodium, it may be as high as 50 grains to the gallon. Should not be more than 3 grains of organic matter destructible by heat, and this mostly vegetable.

3. *Suspicious* when decidedly turbid, even if it be cleared by coarse filtration, or when there is a bad taste or smell. When the mineral matter exceeds 30 grains per gallon (except as above), especially if consisting chiefly of calcium and magnesium sulphates, nitrates, nitrites or chlorides. When, on drying and incineration, there is considerable blackening, or if any quantity of permanganate of potash is readily destroyed, or if the indications of nitrates, nitrites and ammonia are decided. In any of these contingencies water is suspicious.

4. *Impure water*, when so turbid as not to be purified by coarse filtration, or when it has a decided taste. When it contains more than 50 grains of mineral matter, and over four of destructible (presumably organic) matter, or the indications of nitrates, nitrites and ammonia are large. If, on drying and incineration, there is not only great blackening, but decided indications of nitrous acid fumes, or a smell of burnt horn, or if a large amount of permanganate of potash is rapidly decomposed, this water is decidedly impure.

1 and 2 of the above classifications may be used; 3 should be filtered; and 4 should, if possible, be avoided, but if no other be procured, purification should be resorted to.

In contingencies, how shall water be purified? This is an important question, and should be understood by all physicians, for some means of disinfection should be used in all cases where water is suspicious. Any of the following methods may be resorted to and are easily arranged:

If only slightly impure, the addition of 2 or 3 grains of alum to the gallon of water, will materially aid its drinking properties. A sufficient amount of alum should not be used to give a taste of astringency.

If this method is adopted, the water should be allowed to stand for 24 or 36 hours before its consumption.

The addition of permanganate of potash in quantities not sufficiently large to cause discoloration (simply a slight tinge), but which will destroy all impurities, is an excellent method of disinfecting water in small quantities. No definite rule as to the quantity to be used can be given, for the amount required varies with the impurity of the water.

Heat and cold are both good methods of disinfecting, but the water has a flat and disagreeable taste following the use of these agents, due to expulsion of the air it contains. Of the two methods, freezing answers the best purpose, as it expels a larger proportion of the salts.

The best method of purification, and the one adapted for disinfecting large quantities of water, is filtration. For this purpose any porous material may be used—sand, charcoal, sponges, slag wool, etc. Of these charcoal is undoubtedly the best, animal charcoal answering better than vegetable, though either is good. For the purpose of filtration, the powdered charcoal should be tightly packed (not so much as to prevent the water from passing through) in a funnel-shaped vessel and the water poured on. As it slowly trickles from the vessel, it will be found clear, and its taste much improved. One pound of charcoal is sufficient to purify 60 gallons of water, after which the coal should be purified by exposure to a gentle heat, when it may be again used. This substance is supposed to act chemically by means of the large amount of oxygen contained within its meshes.

Many epidemics of some of our worst diseases are due to the constant use of impure water. Especially is this the case in thickly-settled localities or cities, where well water is used, and the soil heavily impregnated with impurities of all kinds. Prominent among the diseases thus caused are diarrhœa and dysentery, cholera, malarial fevers and dyspepsia.

Unquestionably, diarrhœa and dysentery are frequently caused by the impurities of drinking water. The water may contain salts of a purgative character, or some organic impurities may cause the looseness of bowels. The disease may begin as a simple diarrhœa, which may be arrested and the cause removed; or it may continue, to terminate in a graver way, by becoming dysentery. This is undoubtedly the explanation of the great prevalence of these diseases during the summer months, when the conditions necessary for the development of organic impurities are most favorable. These organic impurities are vegetable or animal life of some kind, so minute as to be seen only by the highest power of the microscope. To prove that these impurities do exist, it is only necessary to take a small amount of the purest distilled water, without odor, put into it a small quantity of sediment from the bottom of a cistern or pond, expose to the sunlight of a summer month, and in a few hours the whole will exhibit an exceedingly offensive odor.

Cholera is frequently caused by the use of water contaminated with the excretions of cholera patients. It has even been said that water of itself may cause cholera, but sufficient proof does not exist to allow of this assertion.

The cause of malarial fevers is often traced to the use of impure water. In nearly all epidemics of typhoid fever, its origin has been found in the use of water containing excretions from typhoid patients, or an abundance of putrefying organic matter derived from some other source. Hence, when a patient is afflicted with this malady, care must be taken that the discharges be disinfected, and not deposited near the source of water supply. If there are any large masses of putrefying matter in the neigborhood, they should be removed to prevent contagion from this source.

Yellow fever is caused by impurities both of air and water. Places subject to this fearful plague, should be scrupulously cleansed during the winter months, and great care taken that no impurities accumulate to impregnate the air and water during the months when this fever prevails.

Dyspepsia is, in many instances, caused by the use of impure water. It lessens the secretion of gastric juice; the food thus being left undigested, those unpleasant symptoms, so well known, arise. Many instances are recorded where dyspepsia was prevalent, and by change of water the malady almost totally disappeared. Hard water is more apt to cause this disease, and the change to soft water often benefits.

From the foregoing, it is seen what close relations exist between water, the animal frame and its diseases. The most scrupulous attention should be paid by physicians to this subject, in order to avert those serious calamities which so often bring destruction and misery to various sections of our country. The laity should be informed on this subject, that they may lend their aid for the preservation of their fellow mortals. It should not be the duty of the physician simply to attempt the cure of diseases after they have seized hold on the body, for often the poison is sunk so deep that they are beyond the power of the physician to heal. It is his duty to prevent, as far as in his power lies, those diseases caused by bad hygienic surroundings. Such a true disciple of this noble calling will surely reap his reward; for him *no* reward is too great, no honor too high!

